# Supplementary material for: Combined BSA-Seq and RNA-Seq to Identify Potential Genes Regulating Fruit Size in Bottle Gourd (Lagenaria siceraria L.)
Source: Plants (Basel). 2024 Aug 3;13(15):2154. doi: 10.3390/plants13152154 (PMC11314176; doi:10.3390/plants13152154)
Supplement: Supplementary file 1 [file plants-13-02154-s001.zip › Supplementary Figure.pdf]

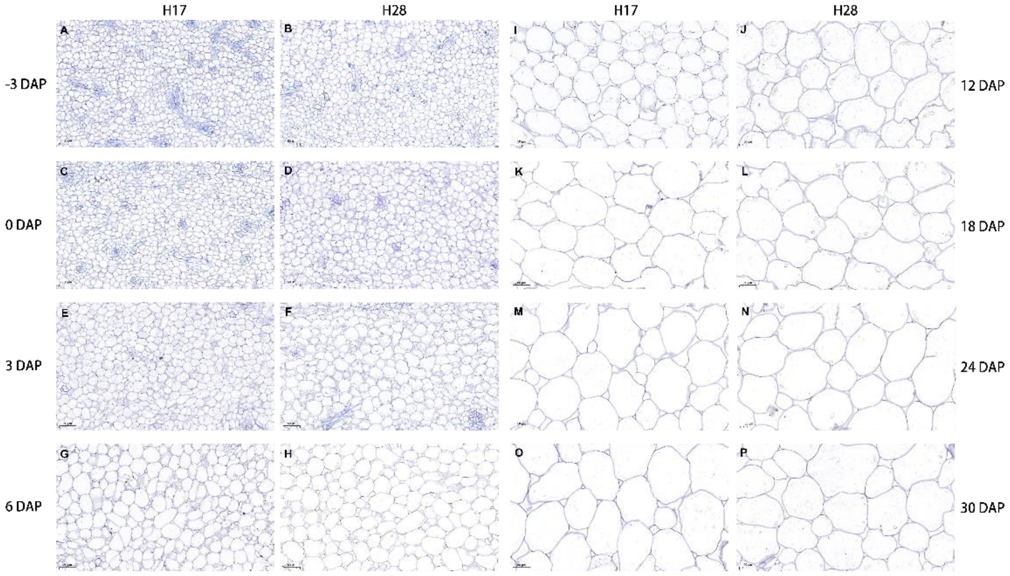

Supplementary Figure S1. Longitudinal section of H17 and H28 fruit at different developmental stages. (A) H17: -3 DAP, (B) H28: -3 DAP, (C) H17: 0 DAP, (D) H28: 0 DAP, (E) H17: 3 DAP, (F) H28: 3 DAP, (G) H17: 6 DAP, (H) H28: 6 DAP, (I) H17: 12 DAP, (J) H28: 12 DAP, (K) H17: 18 DAP, (L) H28: 18 DAP, (M) H17: 24 DAP, (N) H28: 24 DAP, (O) H17: 30 DAP, (P) H28: 30 DAP; Bar = 50 μm.

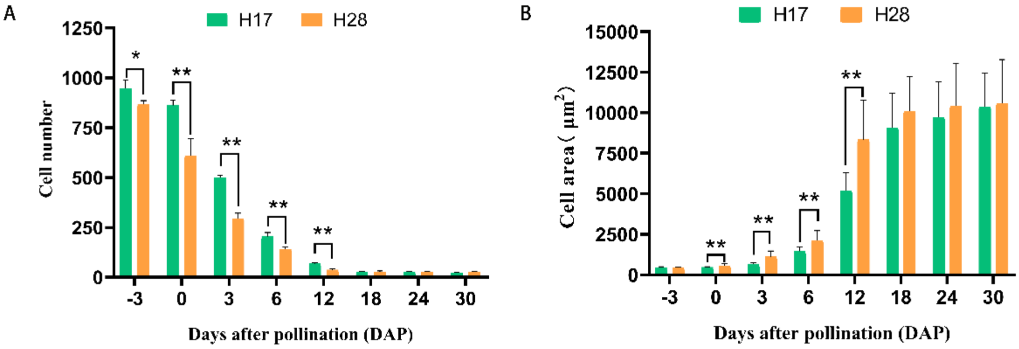

Supplementary Figure S2. Comparison of the average cell area and number in longitudinal sections of H17 and H28 fruit at different periods. (A) Statistical diagram of cell number. (B) Statistical diagram of cell area.
